# Radiofrequency Ablation versus Surgical Resection in Elderly Hepatocellular Carcinoma: A Systematic Review and Meta-Analysis

**DOI:** 10.3390/curroncol31010021

**Published:** 2024-01-06

**Authors:** Jeong-Ju Yoo, Sujin Koo, Gi Hong Choi, Min Woo Lee, Seungeun Ryoo, Jungeun Park, Dong Ah Park

**Affiliations:** 1 Department of Internal Medicine, Soonchunhyang University School of Medicine, Bucheon 14584, Republic of Korea; puby17@schmc.ac.kr; 2 Graduate School of Converging Clinical and Public Health, Ewha Womans University, Seoul 03760, Republic of Korea; 211hcg01@ewhain.net; 3 Department of General Surgery, Yonsei University School of Medicine, Seoul 03722, Republic of Korea; choigh@yuhs.ac; 4 Department of Radiology, Samgsung Medical Center, Sungkyunkwan University, Seoul 06171, Republic of Korea; mw2542.lee@samsung.com; 5 Division of Healthcare Technology Assessment Research, National Evidence-Based Healthcare Collaborating Agency, Seoul 04933, Republic of Korea; ryuu1015@neca.re.kr (S.R.); dubu06@neca.re.kr (J.P.)

**Keywords:** hepatocellular carcinoma, elderly, resection, surgery, radiofrequency ablation

## Abstract

Although the disease burden of elderly cancer patients is rapidly increasing, reliable scientific information, value and preference information of domestic patients, and standardized guidelines for determining the treatment of elderly cancer patients are lacking. The aim of this study is to compare the therapeutic effects of radiofrequency ablation (RFA) and surgery in hepatocellular carcinoma (HCC) patients aged 65 years or older. For the meta-analysis, the databases including PubMed (MEDLINE), EMBASE, OVID Medline, and the Cochrane Library were systematically searched. After the abstract-based review by two investigators, selected manuscripts were read in detail. The surgery group showed higher overall survival (OS) (HR 1.44, 95% CI 1.22–1.70) and disease-free survival (DFS) (HR 1.40, 95% CI 1.00–1.97) than the RFA group. This was also shown in small HCC of less than 3 cm (OS, HR 1.42, 95% CI 1.00–2.03; DFS, HR 1.32, 95% CI 0.91–1.91). This might be related to the high local recurrence in the RFA group (OR 4.90, 95% 2.16–11.08). On the other hand, adverse events were significantly lower in the RFA group (OR 0.22, 95% CI 0.14–0.36), which led to a decrease in the duration of hospital stay (mean difference −14.88 days, 95% CI −22.44–−7.32). In elderly HCC patients, survival in the surgery group was significantly higher than in the RFA group, but various complications tended to increase; so, appropriate patient selection is required.

## 1. Introduction

Hepatocellular carcinoma (HCC), the most common primary malignancy of the liver, contributes to a significant global health burden [1]. With the rising incidence of HCC, it is known to disproportionately occur in the elderly population [2]. As medical advancements and health care improvements extend the average human lifespan, the management of HCC in elderly patients increases in importance and becomes a more complicated clinical challenge. Among the diverse therapeutic modalities available, surgical resection and radiofrequency ablation (RFA) stand out as two prominent approaches for the treatment of early-stage HCC [3].

Surgical resection has been considered to be the gold standard for the curative treatment of HCC, providing the potential for complete tumor removal and improved survival [3]. However, pursuing surgical intervention for elderly patients requires careful consideration due to the higher number of comorbidities, reduced physiological reserves, and increased surgical risks associated with age [4,5]. On the other hand, radiofrequency ablation, a minimally invasive technique, has gained favor as an alternative treatment option. RFA uses thermal energy to destroy tumor tissue and has demonstrated promising outcomes, particularly in patients with small unresectable lesions or those deemed unfit for surgery [6].

In the study of HCC patients without age restrictions, RFA demonstrated non-inferior results compared to surgery, and in particular, the treatment effect of RFA was not different from surgery, especially for early-stage HCCs of less than 2 cm [7,8]. Therefore, RFA became an attractive treatment for elderly HCC patients who were expected to have a greater chance of surgical complications [9]. However, there is no meta-analysis comparing the efficacy and safety of RFA and surgery in elderly HCC patients.

The purpose of this study is to use a systematic review and meta-analysis to comprehensively compare surgical resection and radiofrequency ablation as treatment strategies for elderly patients with HCC. By conducting a systematic review and a meta-analysis of the existing literature, we provide a robust synthesis of the available evidence, shedding light on the relative efficacy, safety, and overall outcomes associated with these interventions in the geriatric HCC population.

## 2. Materials and Methods

The protocol for this review was registered with PROSPERO (International Prospective Register of Systematic Reviews, CRD42023455634) in advance. This systematic review and meta-analysis were performed according to the Preferred Reporting Items for Systematic Reviews and Meta-Analyses (PRISMA) guidelines and the Meta-analysis of Observational Studies in Epidemiology (MOOSE) checklist. Ethics approval was waived from the Institutional Review Board of National Evidence-based Healthcare Collaborating Agency, and the current study conformed to the ethical guidelines of the World Medical Association Declaration of Helsinki.

### 2.1. Study Outcome, Inclusion Criteria, and Exclusion Criteria

The outcome of the study was to compare the efficacy and safety of RFA and surgery in elderly patients with HCC. Efficacy was evaluated using overall survival, disease-free survival, mortality, recurrence, and length of hospital stay, and the safety was evaluated using the incidence of adverse events.

Randomized controlled trials (RCT), prospective, and retrospective cross-sectional or cohort studies were included in the search. We searched for original articles on local ablation therapy or surgical treatment in patients aged 65 or older who were diagnosed with HCC, radiologically or pathologically. The exclusion criteria were as follows: (i) case reports, (ii) case series of fewer than five patients, (iii) review articles, (iv) treatment received other than local ablation or surgery, or (v) papers written in a language other than English or Korean.

### 2.2. Search Strategy 

The search terms included “HCC-related”, “age (elderly)”, and “RFA” or “surgical-related”. We searched for the synonymous terms and used them to develop the search strategies. The keywords used in the Patient/Problem, Intervention, Comparison, and Outcome (PICO) model are listed in the Supplementary Material. We searched Ovid-Medline, Ovid-EMBASE, the Cochrane Central Register of Controlled Trials, KoreaMed, KMBASE, and KISS using Medical Subject Headings terms to identify studies published in English or Korean between 1 January 1974 and 22 March 2022. The search strategies and results of each database search are shown in the Supplementary Material. All search processes were conducted by an expert, Dr. Dong Ah Park. 

### 2.3. Study Selection and Data Extraction

Two authors independently screened the titles and abstracts. Two reviewers (JJY and SJK) independently screened full-text articles for study relevance. Any discrepancy between the two reviewers was resolved by GHC after discussion. Two researchers also independently performed the risk of bias assessment for all included studies. The characteristics and results were extracted and recorded in standard form.

### 2.4. Methodological Quality and the Risk of Bias Assessment

The Risk of Bias Assessment tool for Non-randomized Studies (RoBANS) [10] was used to assess the risk of bias. The overall results are shown in the Supplementary Materials, Risk of Bias section. Any discrepancy between the two authors (GHC and MWL) was resolved by discussion. Publication bias was assessed using funnel plots (Supplementary Figure S1). Publication bias was evaluated only in cases where there were three or more integrated studies. 

### 2.5. Statistical Analysis

The pooled event rate was derived as an outcome of a random-effects model utilizing the following methods: (i) the Freeman–Tukey variant of the arcsine square root transformed proportion was used to convert event rates to proportions; (ii) the Mantel–Haenszel method was used to compute the pooled event rate by back-transforming the weighted mean of transformed event rates. The comparison of the hospitalization dates between two groups was displayed as the mean difference with 95% confidence intervals. We evaluated the inter-study heterogeneity using the I^2^ metric of inconsistency and the *p* value of the Cochran Q test. The I^2^, the ratio of the inter-study variance to the sum of the intra-study and inter-study variance, ranges from 0% to 100%. Publication bias was evaluated by AS-Thompson’s test. Statistical analyses were performed using RevMan 5 (Cochrane Library).

## 3. Results

### 3.1. Characteristics of the Included Studies

After selection, a total of 13 articles were included for final analysis (Supplementary Figure S2). The baseline characteristics of the enrolled studies are presented in Table 1. The final selected papers were all based on retrospective cohort studies, and the total number of patients was 4903. For the standard age threshold for the elderly, 70 years old was the most common age threshold and was found in six articles (46.2%), 65 years old and 75 years old were the age thresholds found in two articles each (15.4%), and 66 years old was the age threshold in one article (7.7%). Also, two studies (15.4%) did not set an age threshold, but the 95% lower limit was 65 years or older. The median follow-up period was 60 months (range 36 to 120 months). When classified by continent, the Asian continent had the most studies with six (46.2%), followed by Europe with five studies (38.5%), and North America with two studies (15.4%). Of the 13 studies, ten studies (76.9%) used the propensity score matching analysis between the two groups, and the remaining three studies (23.1%) did not. Other subject characteristics are presented in detail in the Supplementary Table S1.

### 3.2. Overall Survival

We were able to extract results on the overall survival (OS) from ten articles among the total. The median OS in both groups of the local ablative therapy and resection was 60 months (IQR 45.5–72.4 and IQR 56.0–77.5, respectively) (Supplementary Table S2). The 3-year survival rate was 65.0% (IQR 37.5–68.9) and 70.0% (IQR 61.6–78.5), respectively. Furthermore, the 5-year survival rate was shown by 53.8% and 57.5% (IQR 34.8–55.2% and IQR 50.2–67.5; the local ablative therapy and resection), respectively, demonstrating consistently higher survival rates in the surgery group. 

**Figure 1:**
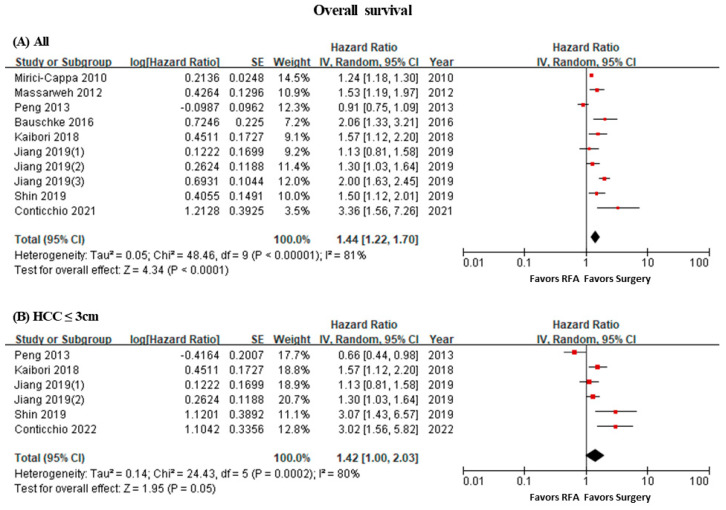
Forest plot of overall survival. (**A**) All, (**B**) sensitivity analysis (≤3 cm).

Comparing the survival rates between the RFA group and the surgery group showed that the survival benefit was significantly higher in in the surgery group than the RFA group, but the heterogeneity between the literature was high (HR 1.44, 95% CI 1.22–1.70, I^2^ = 81%) (Supplementary Table S3, Figure 1A). However, the studies published after 2016 showed decreased heterogeneity (I^2^ = 64%) of the survival benefit in the surgery group. The sensitivity analysis from five articles reporting the results of tumor size ≤ 3 cm demonstrated that the trend of the survival benefit was maintained in the surgical group compared to the RFA group (HR 1.42, 95% CI 1.00–2.03, I^2^ = 80%) (Figure 1B).

### 3.3. Disease-Free Survival

The results of the DFS were obtained from four articles out of the total. The median DFS in the RFA group was 26.3 months (IQR 20.4–42.3), whereas the resection group was 31.0 months (IQR 28.0–57.2) (Supplementary Table S2). Although the 3-year DFS rate did not show a difference between the RFA and surgery groups (62.7% vs. 60.6%), the 5-year DFS rate was significantly higher in the surgery group (30.3% vs. 38.9%).

**Figure 2:**
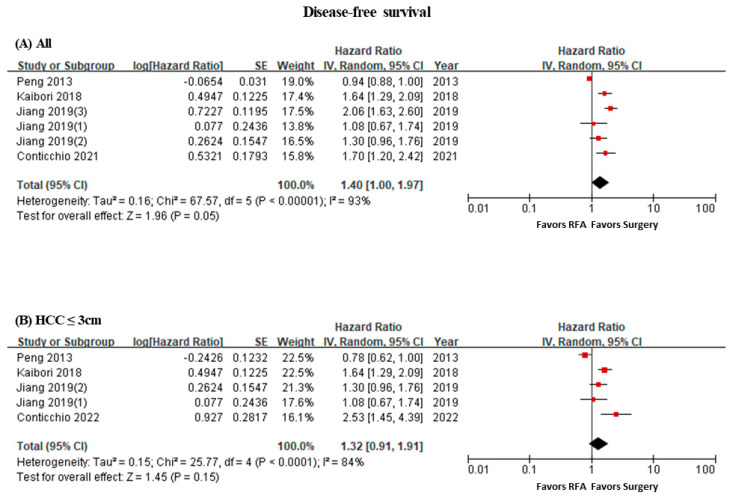
Forest plot of disease-free survival. (**A**) All, (**B**) sensitivity analysis (≤3 cm).

As the meta-analysis on the difference in the DFS between the two groups illustrates, the surgical group had a significantly longer survival (HR 1.40, 95% CI 1.00–1.97, I^2^ = 93%) (Figure 2A). These results are similar to the results of the sensitivity analysis for subjects with a tumor size of 3 cm or less (HR 1.32, 95% CI 0.91–1.91, I^2^ = 84%) (Figure 2B). 

### 3.4. Recurrence

Regarding the recurrence, four articles reported the results by dividing them into total recurrence, local recurrence, intrahepatic recurrence, and extrahepatic recurrence. Local recurrence was the most frequently reported index, which was found in three articles, and the risk of local recurrence was higher in the RFA group compared to the surgical group, which was statistically significant (OR 4.90, 95% 2.16–11.08, I^2^ = 0%) (Supplementary Table S4, Supplementary Figure S3A). The sensitivity analysis of total recurrence, intrahepatic recurrence, and extrahepatic recurrence was reported in two articles, but there was no significant difference between the two groups in the combined results (Supplementary Table S4, Supplementary Figure S3B)

### 3.5. Short-Term Mortality

Mortality was analyzed by dividing it into in-hospital mortality, 30-day mortality, and 90-day mortality. There was no significant difference between the two groups. Hepatic failure-related mortality was reported in two studies, but there was no significant difference (OR 0.09, 95% CI 0.00–1.61) (Table 2). In addition, consistent results were also shown in the analysis of the literature, subject to a sensitivity analysis.

### 3.6. Adverse Events

The overall incidence of AE was reported in three articles, and as a result of the integrated analysis, the RFA group had significantly lower rates of AE than the surgical group; the heterogeneity between articles was low (OR 0.22, 95% CI 0.14–0.36, I^2^ = 0%) (Table 3). In particular, the major complications were significantly lower in the RFA group (OR 0.33, 95% CI 0.13–0.84, I^2^ = 40%). On the other hand, minor complications did not show a difference between groups (OR 1.02, 95% CI 0.11–9.81, I^2^ = 80%). As a result of the individual complication analysis, postoperative hepatic failure, respiratory failure, ascites, hemorrhage, wound site infection, intra-abdominal abscess, and systemic infection showed a lower incidence in the RFA group compared to the surgical group, whereas renal failure, bile leakage, and portal vein thrombosis had no differences between the two treatment groups.

### 3.7. Length of Hospital Stay

There were four articles reporting on the length of hospitalization, and three of them reported that the surgical group had significantly longer stays (Supplementary Table S5). Although the heterogeneity among articles was quite high, the duration of the hospital stay in the RFA group was shorter than in the surgery group by 14.88 days (95% CI −22.44 to −7.32, I^2^ = 99%) (Supplementary Figure S4A). However, a study by Harada et al. reported that there was no significant difference in the length of hospital stay between the RFA group and laparoscopic hepatectomy group. The sensitivity analysis from two articles resulted in a 4.38 day shorter hospital stay in the RFA group than in the surgical group, and there was no heterogeneity between articles (95% CI −5.61 to −3.16, I^2^ = 2%) (Supplementary Figure S4B).

## 4. Discussion

In our study, a meta-analysis was conducted to compare the efficacy and safety of RFA and surgery in elderly patients aged 65 years or older with HCC who lacked information from clinical trials. The comparison of RFA and surgery has been reported by many prospective cohort studies, including RCT, but the results are based on non-elderly patients [24,25]. However, in most clinical trials, there is an age limit for participating in the study due to ethical issues; so, it is difficult to apply the above results to elderly HCC patients. Recently, as the average age of HCC patients is increasing, this meta-analysis can provide treatment evidence that will be applied to clinical practice [26].

The present study found that surgery has a significantly better incidence of overall survival, disease-free survival, and local recurrence than RFA. The dominance of this therapeutic effect was also observed in the sensitivity analysis for small HCC less than 3 cm. The distinction of surgery over RFA in early-stage HCC has been reported in accumulated studies on non-elderly HCC patients. Although the elderly HCC patient has somewhat different characteristics compared to the non-elderly patient, regardless of tumor characteristics or age, surgery showed better results in elderly HCC patients [27,28,29,30]. This might be caused by the nature of the percutaneous approach of RFA, which can be an incomplete treatment if HCC occurs in an area that is difficult to access [31]. In other words, the local recurrence of RFA was increased compared to surgery, leading to a decrease in DFS and overall survival. Since the technology of RFA is developing rapidly, we analyzed publications by year, but a recent study reported a lower OS by RFA compared to surgery. Therefore, apart from the skill of the operator or the development of technology, it is difficult for the RFA technique itself to secure a safety margin as compared to surgery.

The difference between elderly and non-elderly HCC patients is that surgery was consistently superior to RFA for small HCC of less than 3 cm. In non-elderly HCC, there is no statistical difference of OS between RFA and surgery in small HCC of less than 3 cm [32,33]. Therefore, it is better to perform the surgery more aggressively for early-stage HCC in elderly patients, where performance is good and life expectancy is expected to be long. However, the DFS was similar between RFA and surgery up to 3 years, but surgery was superior to RFA after 5 years; so, RFA can be considered for elderly patients with a short life expectancy of less than 5 years.

Our study concluded that RFA is more advantageous than surgery in terms of short-term mortality, postoperative complications, and a shorter hospital stay. This is consistent with previous reports showing the effect in the non-elderly patients with HCC or liver metastasis [34,35]. These studies suggest possible comorbidities in elderly HCC patients. Compared to RFA, surgery has more complications caused by general anesthesia and systemic hemodynamic changes, consequently leading to increased short-term mortality or in-hospital mortality [36,37]. Due to the characteristics of elderly HCC patients, physicians should consider the treatment safety rather than efficacy [9,38]. Although OS or DFS is expected to be limited in elderly HCC patients with high comorbidity, RFA should be actively considered. However, RFA has been reported to induce post-procedural renal failure, bile leakage, or portal vein thrombosis, which are similar to surgery; so, clinical attention is required for these side effects.

The final finding of our study was that RFA shortens the hospitalization period by an average of 14 days compared to surgery. This might be due to the low complication and short-term mortality rates of RFA. In particular, elderly patients are more likely to develop nosocomial infections, sarcopenia, and delirium than non-elderly patients as the hospitalization period increases [39,40]. Ultimately, longer hospital stays lead to increased frailty and medical costs. Therefore, RFA should be considered in high-risk groups who are expected to have a longer hospitalization period through an accurate evaluation of performance and frailty before treatment. However, there have been only limited studies. Harada et al. [17] reported that there was no significant difference in the hospitalization period between the RFA group and the laparoscopic hepatectomy group. Research on laparoscopic surgery should be accumulated in the future [41].

Our study has several limitations. First, since all results are based on retrospective cohort studies, selection bias is inevitable. In particular, it is possible that patients with good performance before treatment were assigned to the surgery group. In this case, additional analysis was performed that adjusted for other factors which affected OS besides treatment, and even after adjustment, surgery was statistically superior to RFA in terms of OS (adjusted HR 1.58, 95% CI 1.43–1.75, Supplementary Figure S5). Second, since the analysis includes both open surgery and laparoscopic surgery, the comparison between laparoscopic surgery and RFA, which are considered to have fewer complications, was not clarified. While our study primarily focuses on comparing RFA with surgical interventions in general for HCC in elderly patients, we acknowledge the importance regarding the differentiation between open and laparoscopic surgical approaches. It is important to highlight that laparoscopic surgery, a form of minimally invasive surgery, is known for its benefits, including reduced morbidity and shorter hospital stays compared to traditional open surgery. These factors, including the Enhanced Recovery After Surgery (ERAS) protocol, are especially relevant in the elderly population, where minimizing complications and recovery time is crucial. Although our current research does not separately analyze open and laparoscopic surgeries, this perspective underlines a valuable aspect of surgical choice in treating HCC. Recently, for HCC located in the anterolateral segments, laparoscopic surgery has been reported to have a similar therapeutic effect and postoperative course to RFA [11]. Future studies could benefit from a more detailed comparison of these surgical techniques, particularly in the context of elderly patients. Third, our study could not evaluate the frailty variable because the studies included in the analysis did not specifically mention factors related to frailty. It seems likely that the elderly patients receiving surgery or RFA treatment for HCC had a relatively good performance status.

## 5. Conclusions

In conclusion, the current meta-analysis study concluded that surgery is superior to RFA in OS, DFS, and local recurrence in HCC patients aged 65 years or older but has disadvantages in terms of postoperative complications, short-term mortality, and length of hospitalization. These results are similar to non-elderly HCC patients, and additional studies targeting elderly patients are expected to be needed in the future.

## Figures and Tables

**Table 1:** The demographics and characteristics of the studies included in the systematic review and meta-analysis.

Study	Country	Study Design	PSM	Participating Institution	Recruitment Period	Inclusion Criteria	Elderly Definition	Number	Intervention Group	Control Group	Follow-Up (Months)
Conticchio 2022 [11]	Europe	Retrospective cohort	Yes	Multicenter	2009–2019	single ≤ 3 cm HCC	70	116 (58/58)	RFA	LLR	36
Conticchio 2021 [12]	Europe	Retrospective cohort	Yes	Multicenter	2009–2019	HCC with Child A-B disease, in BCLC 0/A stage, with tumor within Milan criteria	70	272 (136/136)	RFA	LR	60
Delvecchio 2021 [13]	Europe	Retrospective cohort	Yes	Multicenter	2009–2019	single HCC ≤ 3 cm located in posterosuperior segments (4a, 7, 8)	70	52 (26/26)	RFA	LR	60
Shin 2019 [14]	Korea	Retrospective cohort	Yes	Multicenter	2008–2014	BCLC 0-A staged HCC patients	70	270 (139/131)	RFA	LR	108
Jiang 2019 [15]	USA	Retrospective cohort	Yes	SEER DB	2004–2015	very early- or early-stage HCC	65	1912 (956/956)	RFA	LR	60
Kaibori 2018 [16]	Japan	Retrospective cohort	Yes	Multicenter	2000–2007	early-stage HCC (≤3 cm)	75	922 (461/461)	RFA	LR	60
Harada 2016 [17]	Japan	Retrospective cohort	No	Multicenter	2008–2015	primary HCC with BCLC stage 0 and A disease and portal hypertension	NR	88 (40/48)	RFA	LLR, OLR	84
Bauschke 2016 [18]	Germany	Retrospective cohort	No	Single	1995–2014	HCC patients	70	127 (64/63)	RFA	partial LR	120
Ito 2016 [19]	Japan	Retrospective cohort	Yes	Single	2011–2013	surface HCC (≤3 cm, 1–3 nodules)	NR	54 (27–27)	RFA	LR	36
Liu 2014 [20]	Taiwan	Retrospective cohort	Yes	Single	2002–2013	newly diagnosed HCC	75	257 (139/118)	RFA	LR	120
Peng 2013 [21]	China	Retrospective cohort	No	Single	2003–2007	very early or early HCC (single HCC ≤ 5 cm or up to 3 nodules < 3 cm)	65	180 (89/91)	RFA	OLR	80
Massarweh 2012 [22]	USA	Retrospective cohort	No	Medicare DB	2002–2005	HCC patients	66	415 (206/209)	RFA	LR	60
Mirici-Cappa 2010 [23]	Italy	Retrospective cohort	Yes	Multicenter	1987–2004	HCC patients	70	238 (119/32)	RFA	LR	120

Abbreviations: PSM: propensity score matching, HCC: hepatocellular carcinoma, LR: liver resection, LLR: laparoscopic liver resection, OLR: open liver resection, RFA: radio frequency ablation, NR: not reported, BCLC: Barcelona Clinic Liver Cancer.

**Table 2:** Short-term mortality of the RFA group in comparison with surgical group.

Outcomes	No of Studies	No. of Patients, RFA/Surgery	Pooled OR	95% CI	I^2^	*p* of Chi^2^
**All**						
In-hospital mortality	3	167/217	0.61	0.06–5.93	0%	0.61
30-day mortality	1	206/209	0.51	0.24–1.08	NA	NA
90-day mortality	2	342/345	0.69	0.42–1.14	0%	0.92
Liver failure-related mortality	2	109/159	0.09	0.00–1.61	NA	NA
**Sensitivity analysis: HCC size ≤ 3 cm**						
In-hospital mortality	1	58/58	NA	NA	NA	NA
30-day mortality	0	-	-	-	-	-
90-day mortality	1	58/58	1	0.14–7.35	NA	NA
Liver failure-related mortality	0	-	-	-	-	-

Abbreviations: RFA, radiofrequency ablation; HCC, hepatocellular carcinoma; No., number; NA, non-available; OR, odds ratio; CI, confidence interval.

**Table 3:** Comparison of adverse events in the RFA and surgical groups.

Outcomes	No of Studies	No. of Patients, RFA/Surgery	Pooled OR	95% CI	I^2^	*p* of Chi^2^
Overall complications	3	183/231	0.22	0.14–0.36	0%	0.67
Major complications	3	245/295	0.33	0.13–0.84	40%	0.19
Minor complications	3	183/231	1.02	0.11–9.81	80%	0.006
Postoperative liver failure	3	245/295	0.09	0.02–0.41	0%	0.49
Postoperative heart failure	2	115/117	0.25	0.03–2.28	0%	0.79
Postoperative respiratory failure	1	136/136	0.37	0.14–0.99	NA	NA
Postoperative renal failure	1	136/136	0.16	0.02–1.35	NA	NA
Bile leakage	2	156/204	0.29	0.03–2.49	0%	0.48
Ascites	2	225/227	0.12	0.03–0.42	8%	0.3
Hemorrhage	1	136/136	0.26	0.07–0.94	NA	NA
Wound infection	1	136/136	0.28	0.06–1.35	NA	NA
Intra-abdominal abscess	1	136/136	0.06	0.00–0.97	NA	NA
Portal vein thrombosis	1	136/136	0.5	0.04–5.54	NA	NA
Systemic infection	1	136/136	0.26	0.08–0.82	NA	NA

Abbreviations: No., number; NA, non-available; OR, odds ratio; CI, confidence interval.

## Data Availability

The datasets generated during and/or analyzed during the current study are available from the corresponding author on reasonable request.

## Supplementary Materials

The following supporting information can be downloaded at: https://www.mdpi.com/article/10.3390/curroncol31010021/s1. Table S1: Detailed characteristics of studies included in the systematic review and meta-analysis; Table S2: Comparison of overall survival, disease free survival between surgery and RFA group; Table S3: A meta-analysis of overall survival in the surgical group vs. RFA; Table S4: Comparison of recurrence in RFA and surgical group; Table S5: Comparison of hospital stay in RFA and surgical group; Figure S1: Funnel plots; Figure S2: Flow chart; Figure S3: Forest plot of recurrence rate; Figure S4: Forest plot of hospital stay; Figure S5: Multivariate meta-analysis of overall mortality risk.
