# Supplementary material for: Radiofrequency Ablation versus Surgical Resection in Elderly Hepatocellular Carcinoma: A Systematic Review and Meta-Analysis
Source: Curr Oncol. 2024 Jan 6;31(1):324–34. doi: 10.3390/curroncol31010021 (PMC10813859; doi:10.3390/curroncol31010021)
Supplement: Supplementary file 1 [file curroncol-31-00021-s001.zip › curroncol-2771970-SI.pdf]

Supplementary Material

Table of Contents

I. Supplementary Materials and Methods…………………………………………..……….2
Search strategy and results
Risk of bias
II. Supplementary Table….……………..……………………………….……………..…...8
III. Supplementary Figures….……………..……………………………….……………….14
IV. References………………...……………..……………………………….……………….20

I. Supplementary Materials and Methods

1. Search strategy and results

(1) Summary of search results

No.	DB	Results	Duplication
1	Ovid-MEDLINE	94	
2	Ovid-EMBASE	1159	
3	CENTRAL	113	
4	KoreaMed	53	497
5	KMBASE	34	
6	KISS	313	
Number of Search Results (including duplication)		1766	
**Number of Search Results (without duplication)**		**1269**	

(2) Keywords

(A) International database

1) Ovid-MEDLINE

PICO	No.	Searches
Patients (HCC)	1	exp Carcinoma, Hepatocellular/co, dt, et, mo, rt, su, th [Complications, Drug Therapy, Etiology, Mortality, Radiotherapy, Surgery, Therapy]
	2	((hepat* or liver) adj5 (neoplas* or cancer* or carcin* or tumo?r* or malignan*)).ti,ab.
	3	1 or 2
Patients (elderly)	4	exp Aged/
	5	(advanced years or ageing or aging or aged or elder* or old* or frail* or geriatric* or gerontolog* or oncogeriat* or senior adult* or senium or octogenarian).ti,ab.
	6	4 or 5
Patients	7	3 and 6
Intervention	8	exp Hepatectomy/
	9	(liver or hepat* or segment*) adj5 (surgical or surger* or resect* or trans?ection* or operat* or excis* or extirpate* or segmentectom*).ti,ab.
	10	hepatectom*.ti,ab.
	11	or/8-10
Patients &	12	7 and 11

Intervention		
Patients & Intervention (Human study)	13	Animals/
	14	Human/
	15	13 not 14
	16	12 not 15
SD(RCT) *SIGN filter	17	(Randomized Controlled Trials as Topic/ or randomized controlled trial/ or Random Allocation/ or Double Blind Method/ or Single Blind Method/ or clinical trial/ or clinical trial, phase i.pt. or clinical trial, phase ii.pt. or clinical trial, phase iii.pt. or clinical trial, phase iv.pt. or controlled clinical trial.pt. or randomized controlled trial.pt. or multicenter study.pt. or clinical trial.pt. or exp Clinical Trials as topic/ or (clinical adj trial$).tw. or ((singl$ or doubl$ or treb$ or tripl$) adj (blind$3 or mask$3)).tw. or PLACEBOS/ or placebo$.tw. or randomly allocated.tw. or (allocated adj2 random$).tw.) not (case report.tw. or letter/ or historical article/)
SD (Observational study) *SIGN filter	18	Epidemiologic Studies/ or exp Case Control Studies/ or exp Cohort Studies/ or Case-control.tw. or (cohort adj (study or studies)).tw. or Cohort analy$.tw. or (Follow up adj (study or studies)).tw. or (observational adj (study or studies)).tw. or Longitudinal.tw. or Retrospective.tw.or Cross sectional.tw. or Cross-sectional studies/
SD total	19	17 or 18
P & I (human study) & SD(RCT, cohort)	20	16 and 19
SD (exclusion study design)	21	Case Study/ or case report.tw. or letter/ or Editorial.pt. or review.pt.
Total	22	20 not 21

2) Ovid-EMBASE

	No.	Searches
	1	exp liver cancer/co, dm, dr, dt, et, rt, si, su, th [Complication, Disease Management, Drug Resistance, Drug Therapy, Etiology, Radiotherapy, Side Effect, Surgery, Therapy]
Patients (HCC)	2	exp liver cell carcinoma/co, dm, dr, dt, et, rt, si, su, th [Complication, Disease Management, Drug Resistance, Drug Therapy, Etiology, Radiotherapy, Side Effect, Surgery, Therapy]

	3	((hepat* or liver) adj5 (neoplas* or cancer* or carcin* or tumo?r* or malignan*)).ti,ab.
	4	1 or 2 or 3
	5	exp aged/
Patients (elderly)	6	(advanced years or ageing or aging or aged or elder* or old* or frail* or geriatric* or gerontolog* or oncogeriat* or senior adult* or senium or octogenarian).ti,ab.
	7	5 or 6
Patients	8	4 and 7
Intervention	9	exp liver resection/
	10	exp segmentectomy/
	11	(liver or hepat* or segment*) adj5 (surgical or surger* or resect* or trans?ection* or operat* or excis* or extirpate* or segmentectom*).ti,ab.
	12	hepatectom*.ti,ab.
	13	or/9-12
Patients & Intervention	14	8 and 13
Patients & Intervention (Human study)	15	Animals/
	16	Human/
	17	15 not 16
	18	14 not 17
SD(RCT) *SIGN RCT filter	19	(Clinical Trial/ OR Randomized Controlled Trial/ OR controlled clinical trial/ OR multicenter study/ OR Phase 3 clinical trial/ OR Phase 4 clinical trial/ OR exp RANDOMIZATION/ OR Single Blind Procedure/ OR Double Blind Procedure/ OR Crossover Procedure/ OR PLACEBO/ OR randomi?ed controlled trial$.tw. OR rct.tw. OR (random$ adj2 allocat$).tw. OR single blind$.tw. OR double blind$.tw. OR ((treble or triple) adj blind$).tw. OR placebo$.tw. OR Prospective Study/) not (Case Study/ OR case report.tw. OR abstract report/ or letter/ OR Conference proceeding.pt. OR Conference abstract.pt. OR Editorial.pt. OR Letter.pt. OR Note.pt.)
SD (Observational study) *SIGN filter	20	Clinical study/ OR Case control study OR Family study/ OR Longitudinal study/ OR Retrospective study OR (Prospective study/ not Randomized controlled trials/) OR Cohort analysis/ OR (Cohort adj (study or studies)).mp. OR (Case control adj (study or studies)).tw.OR (follow up adj (study or studies)).tw.OR (observational adj (study or studies)).tw.OR (epidemiologic$ adj (study or studies)).tw. OR (cross sectional adj (study or studies)).tw.
SD total	21	19 or 20
P & I (human study) & SD(RCT, observational)	22	18 and 21

SD(exclusion study design)	23	Case Study/ or case report.tw. or conference abstract.pt. or abstract report/ or letter/ or Editorial.pt. or review.pt.
Total	24	22 not 23

3) CENTRAL (Cochrane Central Register of Controlled Trials)

	No.	Searches
Patients (HCC)	1	MeSH descriptor: [Liver Neoplasms] explode all trees and with qualifier(s): [complications - CO, drug therapy - DT, etiology - ET, mortality - MO, radiotherapy - RT, surgery - SU, therapy - TH]
	2	((hepat* or liver) near/5 (neoplas* or cancer* or carcin* or tumo?r* or malignan*)):ti,ab
	3	[1-#2-#2]
Patients (elderly)	4	MeSH descriptor: [Aged] explode all trees
	5	(advanced years or ageing or aging or aged or elder* or old* or frail* or geriatric* or gerontolog* or oncogeriat* or senior adult* or senium or octogenarian):ti,ab
	6	#4 or #5
Patients	7	#3 and #6
Intervention	8	MeSH descriptor: [Hepatectomy] explode all trees
	9	(liver or hepat* or segment*) near/5 (surgical or surger* or resect* or trans?ection* or operat* or excis* or extirpate* or segmentectom*):ti,ab
	10	hepatectom*:ti,ab
	11	[1-#10-#10]
Patients & Intervention	12	#7 and #11
Patients & Intervention (Human study)	13	MeSH descriptor: [Animals] this term only
	14	MeSH descriptor: [Humans] this term only
	15	#13 not #14
	16	#12 not #15
Total		Trials results

(B) Local database

1) KoreaMed

	No	Searches
Patients	1	(("liver cancer"[ALL])) OR ("hepatocellular carcinoma"[ALL])
	2	(("hepatic"[ALL])) OR ("liver"[ALL])
	3	(("cancer"[ALL]) OR ("neoplasms"[ALL]) OR ("tumor"[ALL]) OR ("carcinoma"[ALL]) OR ("malignant"[ALL]))
	4	2 AND 3
	5	1 OR 4
Patients	6	("ageing"[ALL]) OR ("aging"[ALL]) OR ("aged"[ALL]) OR ("elder"[ALL]) OR ("older"[ALL]) OR ("frail"[ALL]) OR

		("geriatric"[ALL]) OR ("gerontology"[ALL]) OR ("senior adult"[ALL]) OR ("senium"[ALL]) OR ("octogenarian"[ALL])
Intervention	7	("surgical"[ALL]) OR ("surgery "[ALL]) OR ("resection"[ALL]) OR ("operation "[ALL]) OR ("excise"[ALL]) OR ("extirpate"[ALL]) OR ("segmentectomy"[ALL]) OR ("hepatectomy"[ALL])
P&I	8	5 AND 6 AND 7

2) KMBASE

	No	Searches
Patients	1	([ALL=hepatocellular carcinoma] OR [ALL=liver cancer])
Patients	2	(([ALL=aged] OR [ALL=aging]) OR [ALL=elderly]) OR [ALL=older])
Intervention	3	((([ALL=surgery] OR [ALL=resection]) OR [ALL=operation]) OR [ALL=hepatectomy])
P&I	4	1 and 2 and 3
Patients	5	[ALL=간암]) OR [ALL=간세포암종])
Patients	6	((([ALL=노인] OR [ALL=노령]) OR [ALL=고령]) OR [ALL=초고령])
Intervention	7	[ALL=간절제])
P&I	8	5 and 6 and 7
	9	4 OR 8

3) KISS

	No	Searches
Patients	1	전체 = liver cancer|hepatocellular carcinoma|간암|간세포암종
Patients	2	전체 = aging|aged|elderly
	3	전체 = 노인|노령|고령|초고령
Intervention	4	전체 = surgery|resection|operation|hepatectomy|간절제
P&I	5	1 AND 2 AND 4
	6	1 AND 3 AND 4
	7	5 + 6

2. Risk of bias

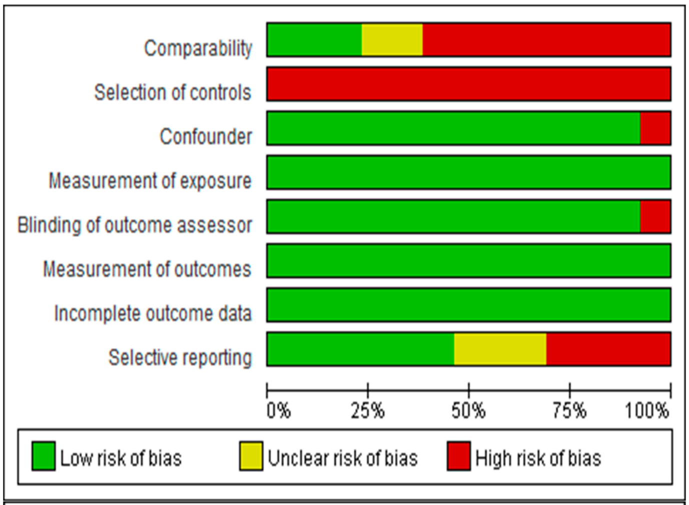

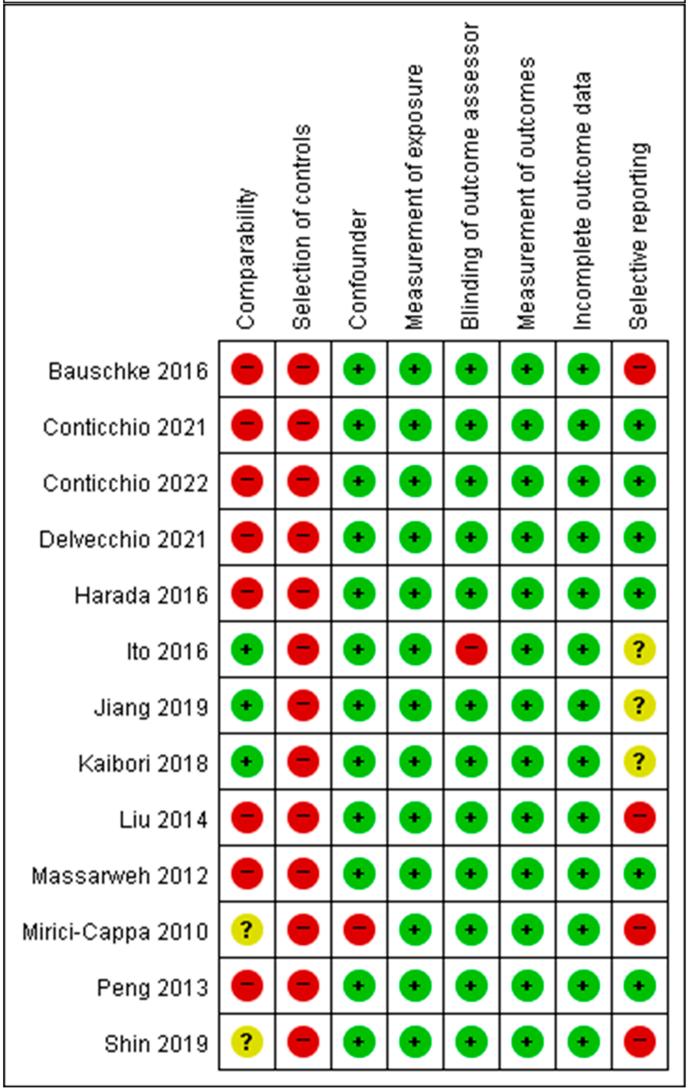

II. Supplementary Tables

Supplementary Table S1. Detailed characteristics of studies included in the systematic review and meta-analysis

Study	RFA									Surgery								
	Intervention	n	Age	Male (%)	BCLC 0/A stage (%)	Child Pugh class A (%)	Tumor size >3cm (%)	Multiple tumors (%)	cirrhosis (%)	Control	n	Age	Male (%)	BCLC 0/A stage (%)	Child Pugh class A (%)	Tumor size >3cm (%)	Multiple tumors (%)	cirrhosis (%)
Conticchio 2022[2]	RFA	58	74	64	NA	90	2.0cm	0	100	LLR	58	75.4	76	NA	86	median 3.0cm	0	100
Conticchio 2021[3]	RFA	136	75	72	100	84	(>2cm) 74	14	100	LR	136	74.7	76	100	85	(>2cm) 79	12	100
Delvecchio 2021[4]	RFA	26	75	65	NA	100	2.05cm	0	100	LR	26	74.26	62	NA	81	median 2.3cm	0	100
Shin 2019[5]	RFA	139	NR	NR	100	NR	NR	NR		SR	131	NR	NR	100	NR	NR	NR	
Jiang 2019[6]	RFA	956	NR	65.6	NR	NR	42	17.7	21.5	LR	956	NR	66.3	NR	NR	45.7	17.2	severe fibrosis or LC 18.9
Kaibori 2018[7]	RFA	461	77	62	NR	89	0	6	NR	LR	461	77	61	NR	89	0	5	NR
Harada 2016[8]	RFA	20	73	55	100	mean 5.5	1.6	mean 1.1	NR	LLR/OLR	20/48	74/70	45/54	100	mean 5.2/5.6	mean 1.8/2.3	mean 1.1/1.3	NR
Bauschke 2016[1]	RFA	64	NR	NR	NA	78	NR	38	77	LR	63	NR	NR	NA	95	NR	22	17
Ito 2016[9]	RFA	27	71	55.6	UICC stage I-II 100	96.3	0	22.2	NR	LR	27	69	59.3	UICC stage I-II 100	96.3	0	7.4	NR
Liu 2014[10]	RFA	139	79.6	67	69.1	64	vol mean 14 cm3	17.3	NR	SR	118	78.9	85	66.7	78.8	vol mean 171 cm3	17.8	NR
Peng 2013[11]	RFA	89	70.4	84.3	NR	87.6	39.2	6.7	100	LR	91	68.7	84.6	NR	94.5	34.1	14.3	100

Massarweh 2012[12]	RFA	206	74.7	63.1	NR	NR	49	44.7	81.6	LR	209	75.7	61.2	NR	NR	80.9	34	49.8
Mirici-Cappa 2010[13]	RFA	119	74.4	68.9	NR	58.8	NR	NR	97.5	LR	32	73.8	71.9	NR	71.9	NR	NR	100

Supplementary Table S2. Comparison of overall survival, disease free survival between surgery and RFA group

Overall survival

	**Overall survival (months)**		**3-year survival rate**		**5-year survival rate**	
	RFA group	Surgery group	RFA group	Surgery group	RFA group	Surgery group
median	60	60	65.0%	70.0%	53.8%	57.5%
range	15.5-85	35.5-88.8	28.8%-78.5%	47.5-80.2%	8.5%-65.5%	37.3%-76.6%

Disease free survival

	**Disease free survival (months)**		**3-year disease free survival rate**		**5-year disease free survival rate**	
	RFA group	Surgery group	RFA group	Surgery group	RFA group	Surgery group
median	26.3	31.0	62.7%	60.6%	30.3%	38.9%
range	20.4-42.3	28.0-57.2	35.7%-75.3%	39.3%-82.0%	16.3%-64.7%	33.1%-77.4%

Abbreviations: RFA: radio frequency ablation

Supplementary Table S3. A meta-analysis of overall survival in the surgical group vs. RFA

Group	Univariable analysis		
	No. of studies	Pooled HR (95% CI) (random model)	I^2
**Overall estimate**	**10**	**1.44 (1.22-1.70)**	**81%**
Using data			
Calculated data	5	1.41 (1.09-1.82)	84%
Reported data	5	1.49 (1.21-1.84)	66%
Definition of elderly			
≥ 65-70 years	9	1.43 (1.20-1.71)	83%
≥ 75 years	1	1.57 (1.12-2.20)	NA
Publication year			
2016-2022	7	1.62 (1.32-1.98)	64%
2010-2015	3	1.19 (0.94-1.50)	85%
Country			
Asia	3	1.26 (0.86-1.87)	84%
Non-Asia	7	1.54 (1.26-1.90)	82%
Risk of bias: comparability			
Low risk	4	1.49 (1.14-1.94)	74%
High or unclear risk	6	1.41 (1.14-1.75)	81%

Abbreviations: No., number; NA, non-available; HR, hazard ratio; CI, confidence interval

Supplementary Table S4. Comparison of recurrence in RFA and surgical group

Outcomes	No of studies	**No. of patients, RFA/surgery**	**Pooled OR**	**95% CI**	I^2	P of Chi^2
**All**						
Total recurrence	2	115/117	0.79	0.47-1.32	0%	0.42
Local recurrence	3	174/176	4.90	2.16-11.08	0%	0.43
Intra-hepatic recurrence	2	147/149	0.62	0.38-1.00	0%	0.64
Extra-hepatic recurrence	2	147/149	0.67	0.11-4.21	0%	0.70
**Sensitivity analysis: HCC size ≤ 3cm**						
Total recurrence	1	26/26	1.17	0.39-3.50	NA	NA
Local recurrence	2	85/85	5.96	1.91-18.64	13%	0.28
Intra-hepatic recurrence	1	58/58	0.52	0.22-1.23	NA	NA
Extra-hepatic recurrence	1	58/58	0.49	0.04-5.57	NA	NA

Abbreviations: HCC, hepatocellular carcinoma; No., number; NA, non-available; OR, odds ratio; CI, confidence interval

Supplementary Table S5. Comparison of hospital stay in RFA and surgical group

Study	Country	Elderly definition	No. RFA/surgery	RFA Median (range), days	Surgery Median (range), days	P
Conticchio, 2022[2]	Europe	70	58/58 (LLR)	3 (1-15)	5 (2-25)	0.001
Conticchio, 2021[3]	Europe	70	136/136 (OLR)	2 (1-23)	7 (1-203)	<0.001
Harada, 2016[8]	Japan	NA	20/20 (LLR)	8 (2-14)	9 (7-14)	not sig
			20/48 (OLR)		15 (7-66)	<0.0001
Ito, 2016[9]	Japan	NA	27/27 (LR)	mean 7.6 (95% CI 6.8-8.4)	mean 12.6 (95% CI 11.0-14.2)	<0.00003
Peng, 2013[11]	China	65	89/91 (LR)	mean 8.01, SD 2.70	mean 13.5, SD 4.05	<0.001

Abbreviations: LLR, laparoscopic liver resection. OLR, open liver resection; RFA, radiofrequency ablation; No., number; NA, non-available; CI, confidence interval; SD, standard deviation

III. Supplementary Figures

Supplementary Figure S1. Funnel plots

(A) overall survival

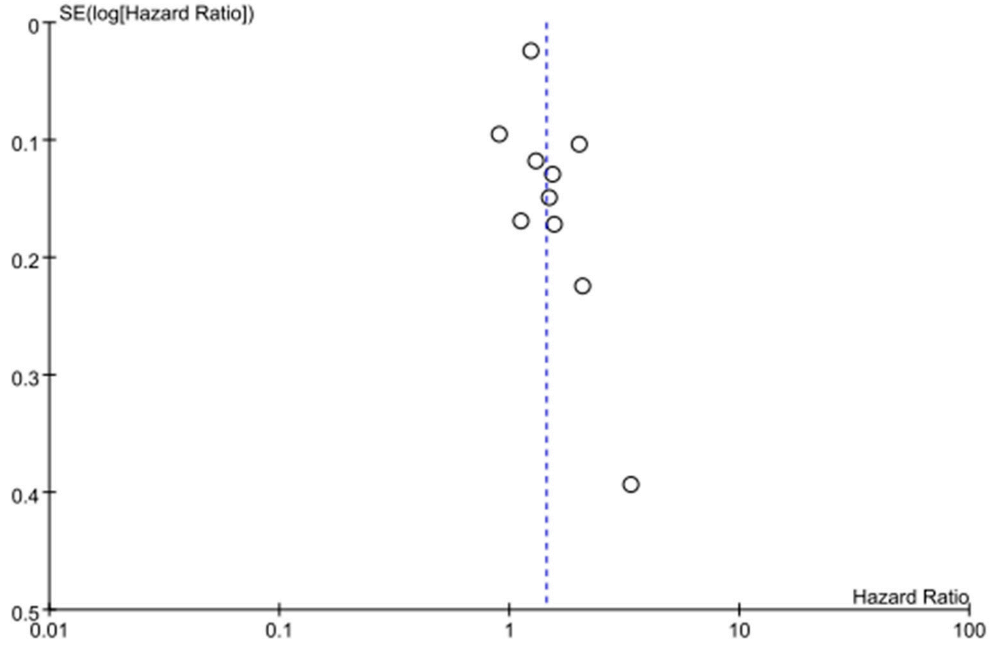

(B) disease free survival

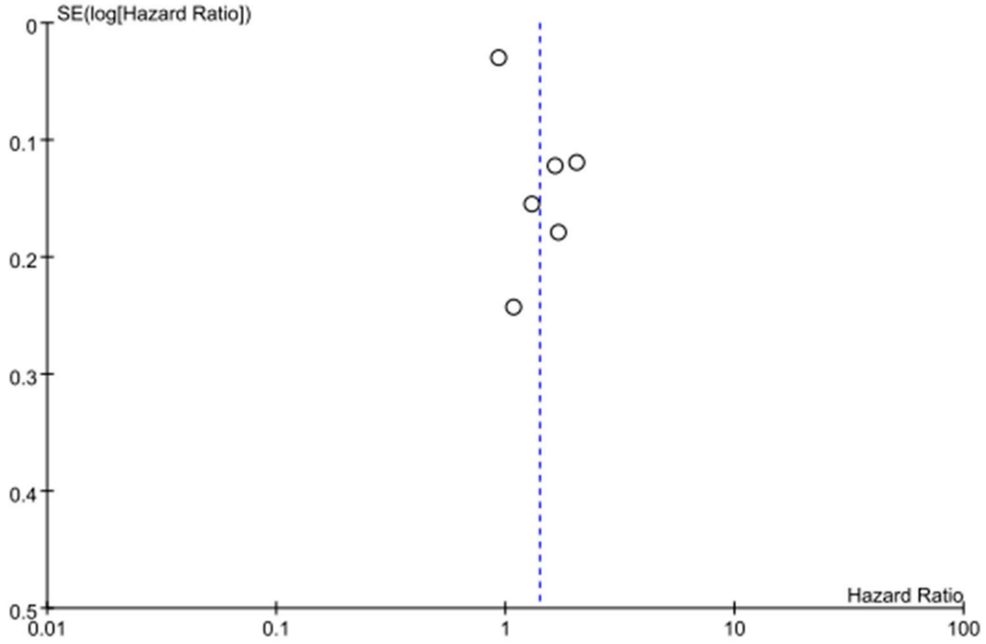

(C) recurrence

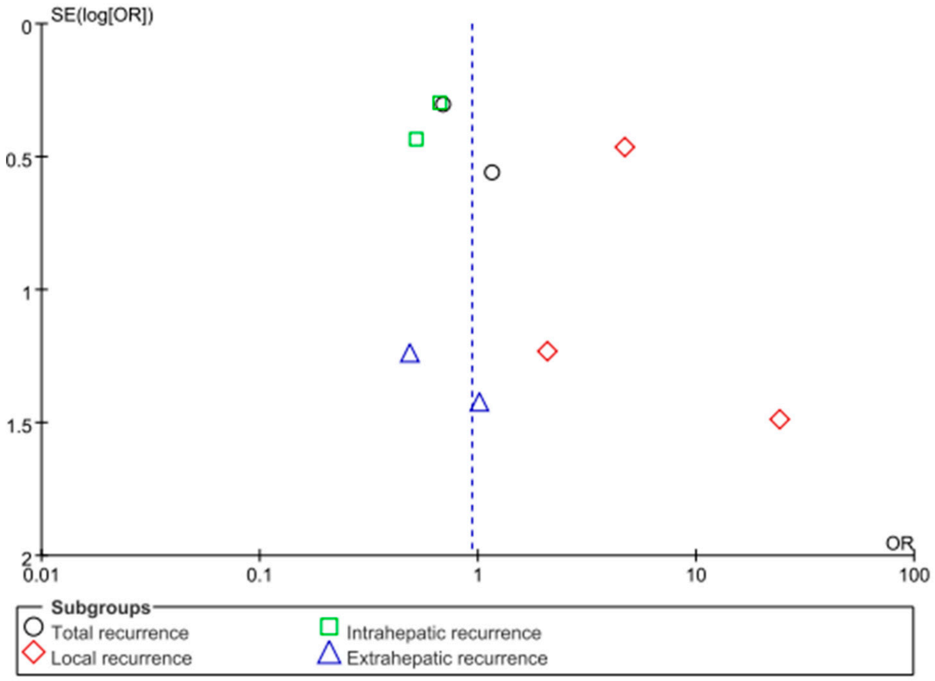

(D) adverse events

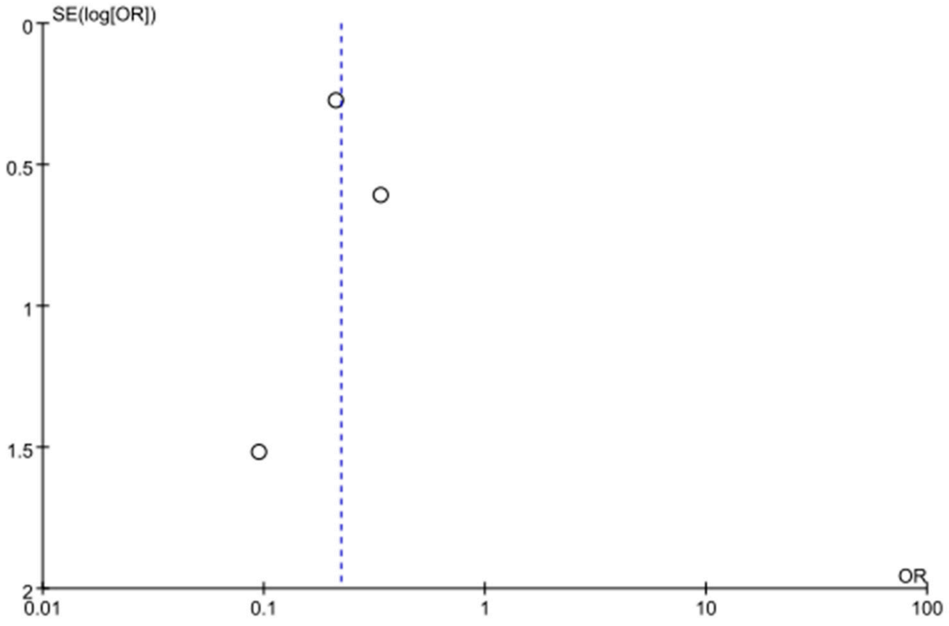

Supplementary Figure S2. Flow chart

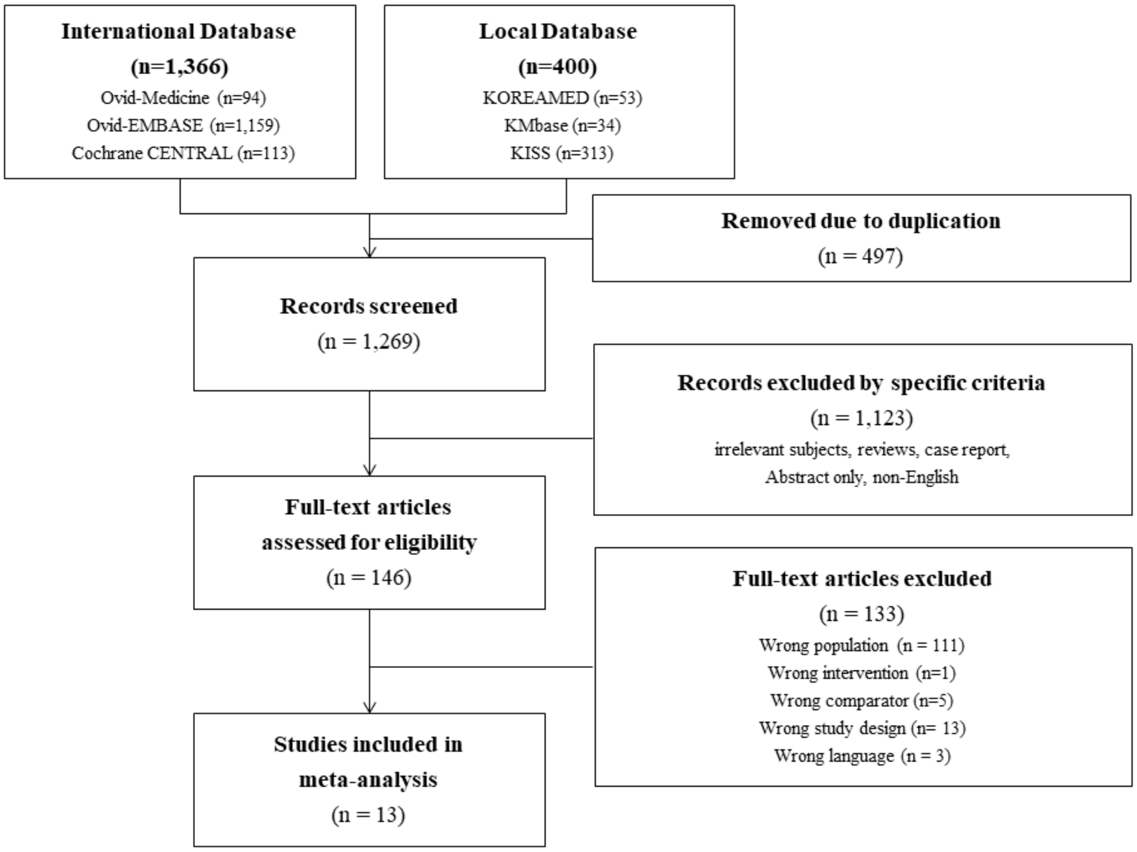

Supplementary Figure S3. Forest plot of recurrence rate
(a) all, (b) sensitivity analysis (≤ 3cm)

Recurrence

Study or Subgroup	LAT Events	LAT Total	LR Events	LR Total	Weight	Odds Ratio M-H, Random, 95% CI	Year
**1. Total recurrence**							
Peng 2013	48	89	57	91	77.2%	0.70 [0.39, 1.27]	2013
Delvecchio 2021	12	26	11	26	22.8%	1.17 [0.39, 3.50]	2021
**Subtotal (95% CI)**		**115**		**117**	**100.0%**	**0.79 [0.47, 1.32]**	
Total events	60		68				
Heterogeneity: Tau² = 0.00; Chi² = 0.66, df = 1 (P = 0.42); I² = 0%							
Test for overall effect: Z = 0.91 (P = 0.36)							
**2. Local recurrence**							
Peng 2013	2	89	1	91	11.4%	2.07 [0.18, 23.23]	2013
Ito 2016	8	27	0	27	7.9%	23.97 [1.31, 440.35]	2016
Conticchio 2022	25	58	8	58	80.7%	4.73 [1.91, 11.76]	2022
**Subtotal (95% CI)**		**174**		**176**	**100.0%**	**4.90 [2.16, 11.08]**	
Total events	35		9				
Heterogeneity: Tau² = 0.00; Chi² = 1.70, df = 2 (P = 0.43); I² = 0%							
Test for overall effect: Z = 3.81 (P = 0.0001)							
**3. Intrahepatic recurrence**							
Peng 2013	45	89	55	91	68.0%	0.67 [0.37, 1.21]	2013
Conticchio 2022	11	58	18	58	32.0%	0.52 [0.22, 1.23]	2022
**Subtotal (95% CI)**		**147**		**149**	**100.0%**	**0.62 [0.38, 1.00]**	
Total events	56		73				
Heterogeneity: Tau² = 0.00; Chi² = 0.22, df = 1 (P = 0.64); I² = 0%							
Test for overall effect: Z = 1.94 (P = 0.05)							
**4. Extrahepatic recurrence**							
Peng 2013	1	89	1	91	43.2%	1.02 [0.06, 16.61]	2013
Conticchio 2022	1	58	2	58	56.8%	0.49 [0.04, 5.57]	2022
**Subtotal (95% CI)**		**147**		**149**	**100.0%**	**0.67 [0.11, 4.21]**	
Total events	2		3				
Heterogeneity: Tau² = 0.00; Chi² = 0.15, df = 1 (P = 0.70); I² = 0%							
Test for overall effect: Z = 0.42 (P = 0.67)							

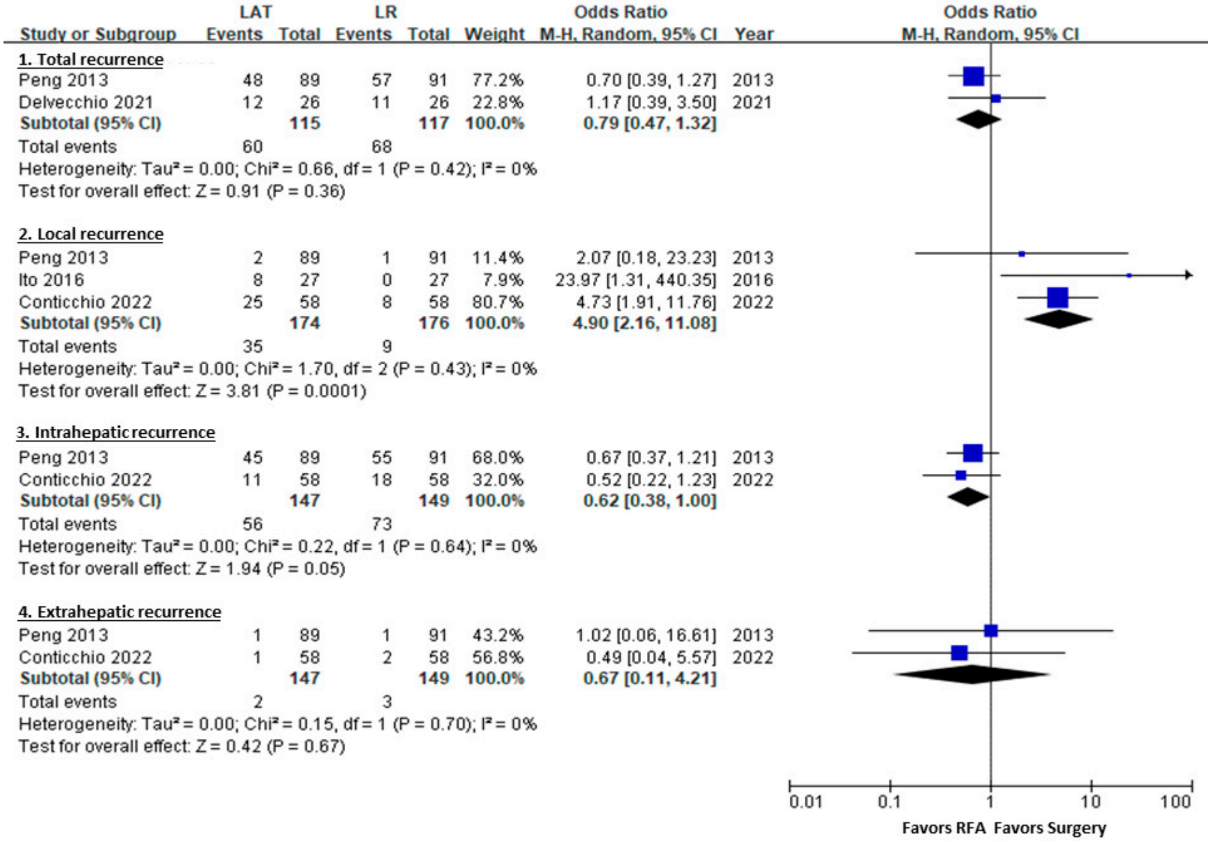

Supplementary Figure S4. Forest plot of hospital stay
(a) all, (b) sensitivity analysis (≤ 3cm)

Length of hospital stay

(A) All

Study or Subgroup	LAT Mean	LAT SD	LAT Total	LR Mean	LR SD	LR Total	Weight	Mean Difference IV, Random, 95% CI	Year
1. Publication year: 2010-2019									
Peng 2013	8.01	2.7	89	13.5	4.05	91	20.6%	-5.49 [-6.49, -4.49]	2013
Harada 2016(2) (1)	8	3	10	25.8	4.75	48	20.2%	-17.80 [-20.09, -15.51]	2016
Ito 2016	7.6	2.0223	27	12.6	4.0446	27	20.4%	-5.00 [-6.71, -3.29]	2016
Harada 2016(1) (2)	8	3	10	9.75	1.75	20	20.3%	-1.75 [-3.76, 0.26]	2016
**Subtotal (95% CI)**			**136**			**186**	**81.5%**	**-7.46 [-12.72, -2.20]**	
Heterogeneity: Tau² = 27.97; Chi² = 120.84, df = 3 (P < 0.00001); I² = 98%									
Test for overall effect: Z = 2.78 (P = 0.005)									
2. Publication year: 2020-									
Conticchio 2021 (3)	7	3.67	136	54.5	33.67	136	18.5%	-47.50 [-53.19, -41.81]	2021
**Subtotal (95% CI)**			**136**			**136**	**18.5%**	**-47.50 [-53.19, -41.81]**	
Heterogeneity: Not applicable									
Test for overall effect: Z = 16.36 (P < 0.00001)									
**Total (95% CI)**			**272**			**322**	**100.0%**	**-14.88 [-22.44, -7.32]**	
Heterogeneity: Tau² = 72.18; Chi² = 319.68, df = 4 (P < 0.00001); I² = 99%									
Test for overall effect: Z = 3.86 (P = 0.0001)									
Test for subgroup differences: Chi² = 102.48, df = 1 (P < 0.00001), I² = 99.0%									

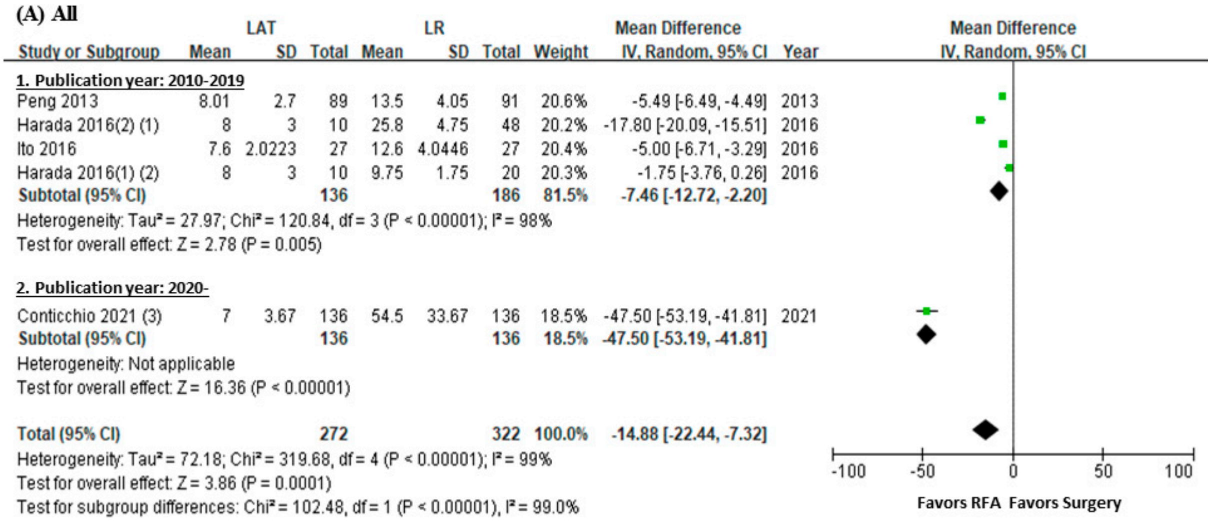

(B) HCC ≤ 3cm

Study or Subgroup	LAT Mean	LAT SD	LAT Total	LR Mean	LR SD	LR Total	Weight	Mean Difference IV, Random, 95% CI	Year
Ito 2016	7.6	2.0223	27	12.6	4.0446	27	50.8%	-5.00 [-6.71, -3.29]	2016
Conticchio 2022 (1)	5.5	3.5	58	9.25	5.75	58	49.2%	-3.75 [-5.48, -2.02]	2022
**Total (95% CI)**			**85**			**85**	**100.0%**	**-4.38 [-5.61, -3.16]**	
Heterogeneity: Tau² = 0.01; Chi² = 1.02, df = 1 (P = 0.31); I² = 2%									
Test for overall effect: Z = 7.02 (P < 0.00001)									

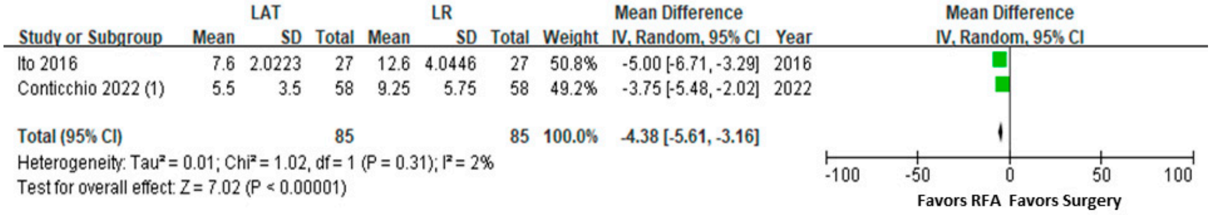

Supplementary Figure S5. Multivariate meta-analysis of overall mortality risk

Study or Subgroup	log[Hazard Ratio]	SE	Weight	Hazard Ratio IV, Random, 95% CI	Year
Peng 2013 (1)	0.6286	0.2331	4.9%	1.87 [1.19, 2.96]	2013
Bauschke 2016 (2)	0.6622	0.3248	2.5%	1.94 [1.03, 3.66]	2016
Kaibori 2018 (3)	0.4253	0.1871	7.6%	1.53 [1.06, 2.21]	2018
Shin 2019 (4)	0.4187	0.1839	7.8%	1.52 [1.06, 2.18]	2019
Jiang 2019 (5)	0.47	0.0681	57.2%	1.60 [1.40, 1.83]	2019
Conticchio 2021 (6)	0.3784	0.115	20.0%	1.46 [1.17, 1.83]	2021
**Total (95% CI)**			**100.0%**	**1.58 [1.43, 1.75]**	

Heterogeneity: Tau² = 0.00; Chi² = 1.52, df = 5 (P = 0.91); I² = 0%
Test for overall effect: Z = 8.87 (P < 0.00001)

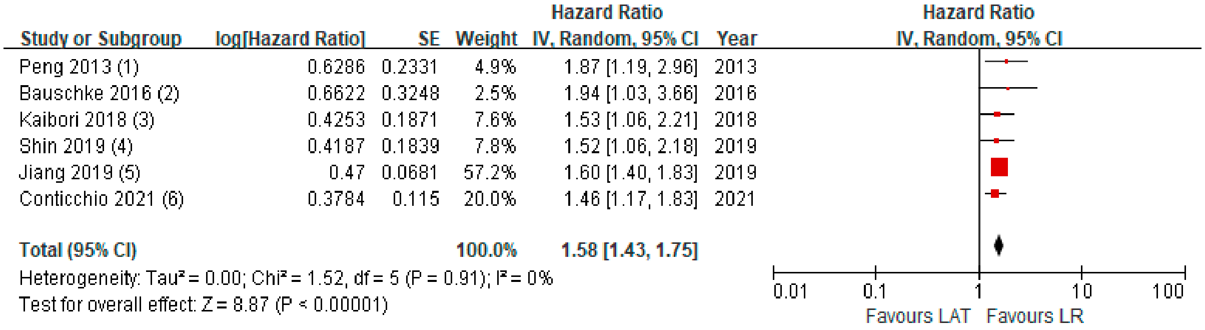

Footnotes
(1) Covariates : Tumor size, prothrombin activity
(2) Covariates : Charlson index, AFP, cirrhosis, Child's stage, Diameter of lesions, Number of lesions
(3) Covariates : age, sex, HBsAg, HCVAb, Child–Pugh class, serum albumin, total bilirubin, prothrombin activity, platelet, a-fetoprotein and...
(4) Covariates : Age, BMI, Albumin, Bilirubin, PT, Creatinine, Tumor number, tumor size, BCLC stages
(5) Covariates : Age, Tumor size, AFP, Fibrosis
(6) Covariates : INR, Multiple nodule, MELD

IV. References

1. Bauschke, A.; Altendorf-Hofmann, A.; Mothes, H.; Rauchfuss, F.; Settmacher, U. Partial liver resection results in a significantly better long-term survival than locally ablative procedures even in elderly patients. *J Cancer Res Clin Oncol* **2016**, *142*, 1099–1108, doi:10.1007/s00432-016-2115-6.
2. Conticchio, M.; Delvecchio, A.; Ratti, F.; Gelli, M.; Anelli, F.M.; Laurent, A.; Vitali, G.C.; Magistri, P.; Assirati, G.; Felli, E.; et al. Laparoscopic surgery versus radiofrequency ablation for the treatment of single hepatocellular carcinoma </=3 cm in the elderly: a propensity score matching analysis. *HPB (Oxford)* **2022**, *24*, 79-86, doi:10.1016/j.hpb.2021.05.008.
3. Conticchio, M.; Inchingolo, R.; Delvecchio, A.; Laera, L.; Ratti, F.; Gelli, M.; Anelli, F.; Laurent, A.; Vitali, G.; Magistri, P.; et al. Radiofrequency ablation vs surgical resection in elderly patients with hepatocellular carcinoma in Milan criteria. *World J Gastroenterol* **2021**, *27*, 2205–2218, doi:10.3748/wjg.v27.i18.2205.
4. Delvecchio, A.; Inchingolo, R.; Laforgia, R.; Ratti, F.; Gelli, M.; Anelli, M.F.; Laurent, A.; Vitali, G.; Magistri, P.; Assirati, G.; et al. Liver resection vs radiofrequency ablation in single hepatocellular carcinoma of posterosuperior segments in elderly patients. *World J Gastrointest Surg* **2021**, *13*, 1696–1707, doi:10.4240/wjgs.v13.i12.1696.
5. Shin, J.; Yu, J.H.; Jin, Y.J.; Suh, Y.J.; Kim, D.H.; Byun, S.; Lee, J.W. Effective therapeutic options for elderly patients with hepatocellular carcinoma: A nationwide cohort study. *Medicine (Baltimore)* **2019**, *98*, e16150, doi:10.1097/MD.0000000000016150.
6. Jiang, Y.Q.; Wang, Z.X.; Deng, Y.N.; Yang, Y.; Wang, G.Y.; Chen, G.H. Efficacy of Hepatic Resection vs. Radiofrequency Ablation for Patients With Very-Early-Stage or Early-Stage Hepatocellular Carcinoma: A Population-Based Study With Stratification by Age and Tumor Size. *Front Oncol* **2019**, *9*, 113, doi:10.3389/fonc.2019.00113.
7. Kaibori, M.; Yoshii, K.; Hasegawa, K.; Ogawa, A.; Kubo, S.; Tateishi, R.; Izumi, N.; Kadoya, M.; Kudo, M.; Kumada, T.; et al. Treatment Optimization for Hepatocellular Carcinoma in Elderly Patients in a Japanese Nationwide Cohort. *Ann Surg* **2019**, *270*, 121–130, doi:10.1097/SLA.0000000000002751.
8. Harada, N.; Maeda, T.; Yoshizumi, T.; Ikeda, T.; Kayashima, H.; Ikegami, T.; Harimoto, N.; Takaki, S.; Maehara, Y. Laparoscopic Liver Resection Is a Feasible Treatment for Patients with Hepatocellular Carcinoma and Portal Hypertension. *Anticancer Res* **2016**, *36*, 3489–3497.
9. Ito, T.; Tanaka, S.; Iwai, S.; Takemura, S.; Hagihara, A.; Uchida-Kobayashi, S.; Shinkawa, H.; Nishioka, T.; Kawada, N.; Kubo, S. Outcomes of laparoscopic hepatic resection versus percutaneous radiofrequency ablation for hepatocellular carcinoma located at the liver surface: A case-control study with propensity score matching. *Hepatol Res* **2016**, *46*, 565–574, doi:10.1111/hepr.12592.
10. Liu, P.H.; Hsu, C.Y.; Lee, Y.H.; Hsia, C.Y.; Huang, Y.H.; Su, C.W.; Chiou, Y.Y.; Lin, H.C.; Huo, T.I. Uncompromised treatment efficacy in elderly patients with hepatocellular carcinoma: a propensity score analysis. *Medicine (Baltimore)* **2014**, *93*, e264, doi:10.1097/MD.0000000000000264.
11. Peng, Z.W.; Liu, F.R.; Ye, S.; Xu, L.; Zhang, Y.J.; Liang, H.H.; Lin, X.J.; Lau, W.Y.; Chen, M.S. Radiofrequency ablation versus open hepatic resection for elderly patients (> 65 years) with very early or early hepatocellular carcinoma. *Cancer* **2013**, *119*, 3812–3820, doi:10.1002/cncr.28293.
12. Massarweh, N.N.; Park, J.O.; Yeung, R.S.; Flum, D.R. Comparative assessment of the safety and effectiveness of radiofrequency ablation among elderly medicare beneficiaries with hepatocellular carcinoma. *Ann Surg Oncol* **2012**, *19*, 1058–1065, doi:10.1245/s10434-011-2067-7.
13. Mirici-Cappa, F.; Gramenzi, A.; Santi, V.; Zambruni, A.; Di Micoli, A.; Frigerio, M.; Maraldi, F.; Di Nolfo, M.A.; Del Poggio, P.; Benvegnu, L.; et al. Treatments for hepatocellular carcinoma in elderly patients are as effective as in younger patients: a 20-year multicentre experience. *Gut* **2010**, *59*, 387–396, doi:10.1136/gut.2009.194217.
